# Local Diversity and Biting Pattern of* Anopheles* Species in Southern Minahasa

**DOI:** 10.1155/2017/6313016

**Published:** 2017-08-06

**Authors:** O. R. Pinontoan, I. G. P. Supadmanaba, I. B. A. Manuaba, I. D. M. Sukrama, I. B. P. Manuaba

**Affiliations:** ^1^Department of Entomology, Graduate Programme, Sam Ratulangi University, Manado, Indonesia; ^2^Biochemistry Department, Faculty of Medicine, Udayana University, Denpasar, Bali, Indonesia; ^3^Department of Medical Education, Faculty of Medicine, Udayana University, Denpasar, Bali, Indonesia; ^4^Department of Microbiology, Faculty of Medicine, Udayana University, Denpasar, Bali, Indonesia; ^5^Biomedicine Postgraduate Program, Udayana University, Bali, Indonesia

## Abstract

**Background:**

To optimize the preventive measures of malaria, it is important to synchronize the efforts with the behavior of local* Anopheles* species. However, the data of* Anopheles* species and their behavior in Indonesia is still lacking.

**Method:**

Explorative research was conducted from April to September 2016 in Southern Minahasa district. The* Anopheles* mosquito was baited by using animal and human (indoor or outdoor) from 18.00 to 06.00 hours. Then, the species were identified and Man Biting Rate (MBR) and Man/Animal Biting per Hour (MBPH) were calculated followed by statistical analysis by using SPSS 17.

**Result:**

The data showed that the dominant species in Southern Minahasa were* An. barbirostris*,* An. kochi*, and* An. vagus*.* An. vagus* was found to be zoophilic and* An. barbirostris* was showing strict anthropophilic characteristics. Meanwhile,* An. kochi* feeds on both human and animal. The MBR of* An. kochi* was found to be the highest (*P* < 0.005), but its MBPH only significantly exceeded that of* An. vagus*. All species tend to be more active during the early evening.

**Conclusion:**

* An. barbirostris*,* An. kochi*, and* An. vagus* were the dominant* Anopheles* species in Southern Minahasa. Further research is needed to determine the* Plasmodium* infestation rate of these species.

## 1. Background

The mosquito is known as a vector for some of the most important human parasites including malaria which is one of important communicable diseases especially in tropical area [1]. Global prevalence rate of malaria was estimated at 214 million cases with 438,000 deaths in 2015 [2]. It is also one of the main burdens of infectious disease in Indonesia with 209,413 new cases in 2015. It is particularly concentrated in Middle and Eastern Indonesia especially in Southern Minahasa which experienced a rise in malarial cases [3].

The diversity of* Anopheles* mosquito poses a real challenge to malaria control programs because different species tend to have different behaviors and feeding locations (indoor versus outdoor) [4]. For example, if predominant* Anopheles* species within the region tend to feed on human outdoor, then common preventive measures like insecticide-impregnated bed nets and indoor spraying will be useless to prevent malaria. Also, the zoophilic tendency is also important in the case of zoophilic-anthropophilic* Anopheles* since the presence of domestic animal near housing will only attract mosquito [5]. Furthermore, this type of* Anopheles* also increases the risk of zoonosis, such as in the case of* P. knowlesi* [6].

Since there are only few studies of* Anopheles* species in Indonesia, the exploration and mapping of* Anopheles* species and its distribution are important. Moreover, Indonesia is an archipelago country with each of its islands or regions having very distinct ecological characteristics from one another; thus it could be hypothesized that the* Anopheles* diversity will also differ from one area to another. So this type of research is urgently needed in Indonesia, especially in malaria endemic areas such as Southern Minahasa.

## 2. Methods

A descriptive explorative research was conducted to identify* Anopheles* species in the region of South Minahasa district and evaluate whether the species are potential vector for malaria from April to September 2016 in South Minahasa district. Sampling was conducted in the areas suspected as* Anopheles* breeding ground for two weeks. Mosquitoes were baited using human and animal and then caught using insect net. The mosquitoes were then placed inside a labeled tube film, identified, and counted. The protocol of using human and animal bait is described as follows.

### 2.1. Human Bait

The mosquitoes were lured by using four people as human baits who worked in shifts between 18.00 and 06.00 hours. As a preventive measure, malaria prophylaxis (chloroquine) had been given one week before research and continued for up to 1 month after the end of the study. Sample collection was conducted both inside and outside the house from families that had at least one member with malaria. The human bait wore a short and rolled up the cloth sleeve, exposing the whole arm to the mosquito. The human bait was also asked not to smoke during the baiting period.

### 2.2. Animal Bait

Mosquito catching using animal baits was conducted in the animal pen and its surrounding areas with no special treatment given to the animal. All mosquitoes that landed on the animal were collected using aspirator and stored inside labeled container.

All collected specimens were identified using taxonomical book by O'Connor and Soepanto (1999) [7].

The density of mosquitoes that contact the human and animal bait was calculated by using following equation.

(a) The Man Biting Rate (Man Biting Rate) [8]:
  (1) $$\begin{array}{c} \frac{the\;\;number\;\;of\;\;mosquitoes\;\;on\;\;human\;\;bait\;\;per\;\;night}{the\;\;number\;\;of\;\;catching\;\;attempts\times period\;\;of\;\;catching\;\;activity}. \end{array}$$
(b) Man/Animal Biting per Hour (MBPH) [9]:
  (2) $$\begin{array}{c} \frac{the\;\;number\;\;of\;\;mosquitoes\;\;on\;\;human\;\;bait\;\;per\;\;hour}{the\;\;number\;\;of\;\;catching\;\;attempts\times period\;\;of\;\;catching\;\;activity}. \end{array}$$

All data were analyzed descriptively to see the diversity of the species, the pattern of mosquito density, and its biting pattern. All data were analyzed by normality test (Shapiro-Wilk) to determine the distribution pattern. Next, we compared the density and biting pattern of each species to evaluate whether the differences were statistically significant. All analyses were conducted with SPSS.17 for Windows.

## 3. Results

### 3.1. Diversity Profile of* Anopheles* Mosquito Caught in Southern Minahasa

Table 1 describes the profile of* Anopheles* species that was found in Southern Minahasa as well as their respective frequency from May to September 2016.

It appeared that* An. barbirostris* and* An. parangensis* were the most common species inside the house. Meanwhile,* An. parangensis *and* An. maculatus* were more common outside the house. It was interested that* An. parangensis* was found to be quite common both inside and outside the house. No* An. vagus* or* An. kochi* was found in human bait experiment, but both of them were commonly found in animal bait experiment.

### 3.2. The Density and Biting Pattern of* Anopheles* Mosquito in Southern Minahasa

Next, we calculated the MBR of* An. vagus, An. kochi*, and* An. barbirostris*. The result of MBR of those species is described in detail in Tables 2, 3, and 4. Because* An. vagus *feeds on animals, we calculate its MBR and MBPH based on the number of mosquitoes found feeding on domestic animals.  Table 2 shows that the MBR of this species ranges from 0.3 to 0.9 mosquitoes per animal. Meanwhile, evaluation of MBPH revealed that the feeding activity of* An. vagus *took place between 18.00 and 22.00 hours with a momentary pause between 22.00 and 05.00 hours. Then, the feeding activity resumed briefly from 05.00 to 06.00 hours. The MBPH of this species ranged from 0.1 to 0.2 mosquitoes/animal/hour.

We evaluated the same variable* for An. kochi*. It appears that the MBR of* An. kochi* in animals ranges from 0.31 to 1.64, which was higher than that of* An. vagus*. Also, its MBPH ranged from 0.02 to 0.35 inside the house and 0.02 to 0.54 outside the house. MBPH data indicate that the feeding activity of* An. kochi* began as early as 06.00 a.m. and rose sharply in the next 2-hour period. Meanwhile, it decreased steadily for the next 2 hours until there was no detectable activity between 02.00 and 04.00 hours. Then, there was some feeding activity during the last 2-hour period. In contrast, the activity of* An. kochi* outside the house was much higher during the first 6 hours. Later on, it decreased sharply and stabilized at 0.02 mosquitoes/man/hour. In contrast to* An. vagus, An. kochi* is known to feed on both humans and animals, so the calculation of MBPH was focused on human bait.

We evaluated the corresponding variables for* An. barbirostris*, which is known to feed only on human. Compared to* An. vagus* and* An. kochi, An. barbirostris* has higher rate of MBR ranging from 0.71 to 0.89 inside the house and 0.11 to 0.45 outside the house. Meanwhile, its MBPH was not so different than the other two. The MBR rate inside the house was also higher than the outside; meanwhile, the opposite was observed in MBPH rate for the first 5 hours. Then, the MBPH rate began to fall and stabilized at 0.02–0.04 mosquitoes/man/hour. Interestingly, no activity was observed between 01.00 and 04.00 hours.

### 3.3. Comparison of MBR and MBPH between* An. vagus*,* An. kochi*, and* An. barbirostris*

Lastly, we compared both variables (MBR and MBPH) between* An. vagus, An. kochi*, and* An. barbirostris*. The details of comparisons are presented in Tables 4 and 5. From MBR perspective, it was clear that the exophytic density of* An. vagus* and* An. kochi* exceeded that of* An. barbirostris* significantly. In addition, the endophytic density of* An. barbirostris* also exceeded its exophytic density.

Meanwhile, from MBPH data, it was clearly seen that only the difference between MBPH of* An. vagus* and exophytic* An. kochi* was statistically significant (Table 5). Considering that all species tend to be more active during the first half of the night, we decided to exclude the other 6-hour periods of observation from analysis (*reanalysis*; Table 5). On reanalysis, it appeared that the endophytic and exophytic activity of* An. kochi* were significantly different. Meanwhile, no significant difference was observed between exophytic and endophytic* An. barbirostris*.

## 4. Discussion

Malaria is one of the most deadly and widespread parasitic disease in the world [2]. Many attempts had been made to control, prevent, and even eliminate the disease. One of them is by studying the species diversity and behavior of* Anopheles* mosquito, the vector of malaria [4]. This field of research is considered to be important because it could identify the specific species of* Anopheles* responsible for malaria transmission from human to human or animal to human and vice versa [6]. It could also contribute to malaria prevention by matching the preventive programs to predominant* Anopheles* species within the area [4].

There are several interesting facts that we obtained from this exploratory study. We found there were five species of* Anopheles* in the Southern Minahasa region:* An. barbirostris, An. parangensis, An. maculatus, An. vagus*, and* An. kochi*. The first three species were found to feed on humans; meanwhile, the other two were found to only feed on animals. Meanwhile,* An. kochi* was found to feed on both humans and animals. The frequency of each species is shown in Table 1.

Our study results clearly showed that* An. barbirostris* and* An. kochi* were the predominant species. This finding is different from that reported in studies conducted in other locations in Southeast Asia, such as Malaysia, Thailand, Vietnam, and Lao PDR, where it was reported that* An. maculatus* is the dominant species [6, 10–12]. Similarly, Sumba Island, which is also in the Central Indonesia region, is dominated by another species, namely,* An. sondaicus* [13]. However, our results are indeed consistent with studies conducted by Ndoen et al. and Pinontoanetal (unpublished), but* An. kochi* was not considered to be a common species by both studies [14, 15]. The reason for the discrepancy might be because the population of* An. barbirostris, An. parangensis*, and* An. maculatus* tends to be higher in populated areas, especially inside the house, which increases the chances of catching in this study [4, 14]. Meanwhile, this study focused on the peripheral area which is less populated but close to shrub and jungle areas which are suitable breeding grounds for* Anopheles*. Therefore, the other two species were also caught.

By comparing their MBR and MBPH rates, all the analyzed species were considered potential vectors because their MBR rates exceeded the threshold level for vector, which is 0.025 mosquitoes/human/night [16]. However,* An. vagus* fed specifically only on domestic animals and showed no interest toward human. The preference of* An. vagus* for animals to humans was also reported by several other researches [5, 14, 17, 18]. However, it had also been identified as malaria vector in some occasions, especially during outbreaks [19]. Furthermore, it was also found to bite people who live close to the cattle pen [18].

On the other hand,* An. barbirostris* was found to specifically feed on humans, which was confirmed by the absence of this species during the animal bait experiment. The density (MBR) of this species was higher than that of* An. vagus* but lower than that of* An. kochi*, though the difference between it and* An. vagus* was not statistically significant. It was also found both inside and outside the house, but its endophytic MBR was significantly higher, which suggests that this species was more active indoor. Based on the pattern of its MBPH, it was found that* An. barbirostris* tended to remain active during the first 4-5 hours of the evening. However, the MBPH pattern showed that this species was slightly more active outdoor.* An. barbirostris* is widely distributed in Southeast Asia and some studies had also confirmed it as a* Plasmodium* vector [20, 21]. However* An. barbirostris* was never found to be a common species in other studies conducted in Southeast Asia and its density had always exceeded the density of* An. maculatus, An. sondaicus, *and* An. dirus* [6, 10]. Furthermore, Amerasinghe et al. and Reid et al. reported that it is the anthropophilic-zoophilic type which is contrary to our findings [22, 23].

As for the comparison of the last two* Anopheles* species, the feeding pattern of* An. kochi* was found to be similar to that of* An. barbirostris*, but* An. barbirostris* was found feeding on both humans and animals. By comparing its MBR and MBPH, it was clear that the density of this species exceeded the densities of the other two species. Furthermore, its exophytic feeding activity was also higher than its endophytic activity, although there were no statistically significant differences between its MBPH and that of* An. vagus* and exophytic* An. barbirostris*. This species tends to be more widely distributed compared with other* Anopheles* species, which is confirmed by Pinontoan [15]. The same study had also stated that this species was invested with* Plasmodium*, which highlights the potential role of this species in malaria transmission. However, a study conducted in Maluku region of eastern Indonesia by Soekirno et al. reported that* An. kochi* was not infectious and did not act as an effective vector for* Plasmodium* [24]. Jiram et al. also studied this species to find out whether it was susceptible to animal* Plasmodium* infection but could not find any sporozoite [6]. Thus, it remains unclear whether this species could act effectively as a* Plasmodium* vector and further researches are needed to confirm the findings reported* by Pinontoan* [15].

Overall, our findings reveal the local diversity in Southern Minahasa region of Celebes Island, Indonesia. This finding was different compared with the other studies conducted in Southeast Asia. In this study, the density of* An. maculatus* was found to be lower than that of the other four species; meanwhile, it was found to be predominant in several countries of Indochina peninsula [4, 10–12]. Furthermore,* An. farauti* and* An. punctulatus* are predominant in Papua New Guinea and* An. sondaicus* is commonly found in Sunda Island, Indonesia [13, 25, 26]. It seems there is great diversity in species that contributes to malaria transmission between different areas in Southeast Asia. However, all studies stated that* Anopheles* mosquito tends to be more active during the first hour of the evening, which is in accordance with the findings of this study. Nevertheless, this study provided additional data about* Anopheles* species in Indonesia which still has high prevalence of malaria.

## 5. Conclusion

Based on the results of this study, we found that* An. kochi* and* An. barbirostris* were the main vectors of malaria in Southern Minahasa district, with* An. barbirostris* feeding specifically on humans and* An. kochi* feeding on both humans and animals. It also appears that they tend to be more active during the first hour of the evening whereas their activity drops significantly later on. In regard to* An. kochi*'s feeding behavior, it can be concluded that having a domestic animal around the settlement could attract this species and, hence, increase the risk of malaria infection. However, further research is needed to investigate the* Plasmodium* infestation rate among these* Anopheles* species in order to complete the map of species distribution and susceptibility areas in Minahasa Region and Indonesia.

## Figures and Tables

**Table 1:** Profile of *Anopheles* mosquito species and the average number of each species with human and animal baits.

Period of catch	Human bait						Animal bait	
	Indoor			Outdoor				
	(1)	(2)	(3)	(1)	(2)	(3)	(4)	(5)
May	17	4	17	9	14	20	14	14
June	19	17	7	7	26	17	22	20
July	20	29	20	3	28	19	14	46
August	27	30	5	4	39	16	16	19
September	32	14	4	5	32	14	20	11

*Average*	23	18.8	10.6	5.6	27.8	17.2	17.2	22

*Note*. (1) *An. barbirostris*; (2) *An. parangensis*; (3) *An. maculatus*; (4) *An. vagus*; (5) *An. kochi*.

**Table 2:** Man Biting Rate (MBR) of *An. vagus*, *An. kochi*, and *An. barbirostris*.

Month	Man Biting Rate (MBR)			
	*An. vagus*	*An. kochi*	*An. barbirostris*	
	Animal bait	Animal bait	Exophytic human bait	Endophytic human bait
May	0.7	0.7	0.45	0.85
June	0.9	0,83	0.29	0.79
July	0.5	1.64	0.11	0.71
August	0.3	0.59	0.13	0.84
September	0.6	0.31	0.14	0.89

**Table 3:** Man/Animal Biting per Hour of *An. vagus*, *An. kochi*, and *An. barbirostris*.

Timing period	Man/Animal Biting per Hour (MBPH)					
	*An. vagus*		*An. kochi*		*An. barbirostris*	
	Exophytic human bait	Endophytic human bait	Exophytic human bait	Endophytic human bait	Exophytic human bait	Endophytic human bait
18.00–19.00	0.1	0	0.29	0.02	0.31	0.17
19.00–20.00	0.2	0	0.46	0.35	0.56	0.35
20.00–21.00	0.2	0	0.54	0.29	0.35	0.15
21.00–22.00	0.1	0	0.39	0.14	0	0.04
22.00–23.00	0	0	0.39	0.10	0.21	0.02
23.00–24.00	0	0	0.33	0.06	0.04	0.02
00.00–01.00	0	0	0.13	0.04	0.04	0.02
01.00–02.00	0	0	0.02	0.02	0.02	0
02.00–03.00	0	0	0.02	0	0.02	0
03.00–04.00	0	0	0.02	0	0.02	0
04.00–05.00	0	0	0.02	0.02	0	0.04
05.00–06.00	0.1	0	0.02	0.08	0.08	0.08

**Table 4:** MBR comparison between *An. vagus, An. kochi*, and* An. barbirostris*.

Species	Species comparison	*P* value
*An. vagus *	*An. kochi *	0.53
	Endophytic *An. barbirostris *	0.073
	Exophytic *An. barbirostris *	0.014

*An. kochi*	Endophytic *An. barbirostris *	0.251
	Exophytic *An. barbirostris *	0.016

Endophytic *An. barbirostris *	Exophytic *An. barbirostris *	

**Table 5:** Comparison of MBPH between *An. vagus,  An. kochi*, and *An. barbirostris*.

Species	Species comparison	Normality test (Shapiro-Wilk)	*P* value	*P* value (Reanalysis)
*An. vagus *	Exophytic *An. kochi *	0.499	0.008^*∗*^	0.000^*∗*^
	Exophytic *An. barbirostris *	0.196	0.137	0.149
Endophytic *An. kochi *	Exophytic *An. kochi *	0.655	0.158	0.004^*∗*^
	Endophytic *An. barbirostris *	0.185	0.619	0.651
Exophytic *An. kochi *	Exophytic *An. barbirostris *	0.169	0.331	0.126
Endophytic *An. barbirostris *	Exophytic *An. barbirostri*s	0.177	0.464	0.258

^*∗*^ *Reanalysis*. We only used MBPH data that pertained to the period 18.00–24.00 hours.
